# Mesenteric lymphatic hygroma in adults: A case report with a review of the literature

**DOI:** 10.3892/ol.2013.1778

**Published:** 2013-12-27

**Authors:** YI YANG, YANTAO CAI, ZHENYANG LI, YANTIAN FANG, JIANBIN XIANG, ZONGYOU CHEN

**Affiliations:** Department of General Surgery, Huashan Hospital Affiliated to Fudan University, Shanghai 200040, P.R. China

**Keywords:** lymphatic hygroma, mesentery, surgery, adult

## Abstract

Mesenteric lymphatic hygroma is a benign tumor of lymphatic origin that is rarely observed in adult patients. Congenital and developed non-specific symptoms, including abdominal distension, pain and ileus, develop at an early age in patients. This type of disease is usually reported by pediatric doctors, as referred to in the literature. The current study presents the case of a 23-year-old male in whom a polycystic mass in the mesentery was identified by computed tomography. The size of the tumor was measured to be 30×20×15 cm during surgery. The mass was excised completely with preservation of the intestine, duodenum, pancreas and other neighboring organs. Histopathological examination confirmed the diagnosis of lymphatic hygroma. The post-operative recovery was uneventful, with the exception of chylous leakage for one week, which was relieved spontaneously. In addition, the present study presents a review of the previous literature concerning mesenteric lymphatic hygroma.

## Introduction

Lymphatic hygromas are rare lymphatic vessel malformations, which are localized in areas of abnormal development of the lymphatic system. Commonly, the disease is diagnosed during childhood, is located in the head and neck region and occurs in one out of every 2,000–4,000 live births (1,2). Only 5% are found in the abdominal cavity, including the small intestine, colon and mesentery (2–4). Lymphatic hygromas predominantly present in childhood, with >50% diagnosed at birth and 90% by 2 years of age (5,6). However, a large number of abdominal lymphatic hygromas do not manifest until adulthood (7). Proper diagnosis remains difficult for abdominal lymphatic hygroma during routine examinations, particularly in adults. Further progression in standard therapy is necessary for improving treatment of all lymphatic hygromas. The current study presents the case of a male adult with mesenteric lymphatic hygroma and a literature review of lymphatic hygroma.

## Case report

**Figure 1:**
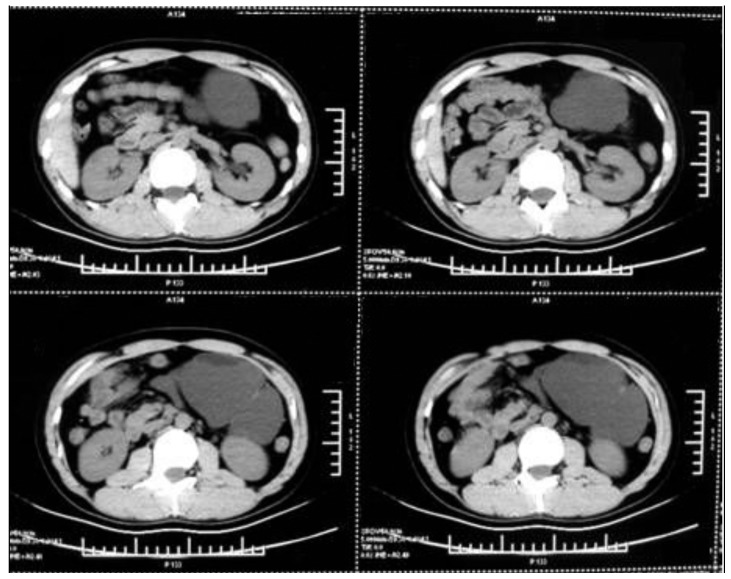
Computed tomography scan indicating a huge cystic mass in the midabdomen, with an estimated maximum diameter of >15 cm.

A 23-year-old male was admitted to Huashan Hospital Affiliated to Fudan University (Shanghai, China) with the chief complaint of progressive dull pain in the upper abdominal region that had been present for seven years. This symptom worsened when the patient was lying down, particularly in a lateral position, and was slightly relieved by a horizontal position. In addition, the patient suffered from a change in bowel habits with an irregular alternation between diarrhea and constipation. The patient did not receive any related medical examinations until seven days prior to admission. A computed tomography (CT) scan indicated a huge cystic mass in the midabdomen, with an estimated maximum diameter of >15 cm (Fig. 1). The top three suspected radiological diagnoses were teratoma, lipoblastoma and lymphatic hygroma. A physical examination revealed a huge mass with soft feedback and a vague boundary upon palpation. No other significant features were found.

**Figure 2:**
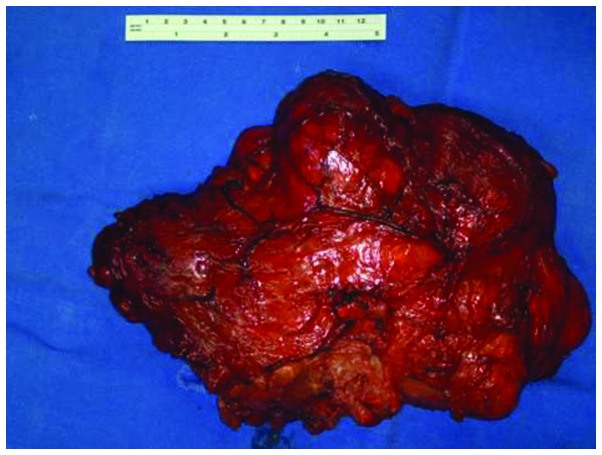
Cystic mass following surgery, with an estimated maximum diameter of >20 cm.

A laparotomy was performed with a median abdominal incision. Intraoperative exploration revealed a huge mass within the small bowel mesentery extending into the mesentery of the left colon. The size of the mass was measured to be ~30×20×15 cm. The appearance of the mass was found to be yellowish, cystic and lobulated. The capsule was almost complete, with the exception of an unclear section of the Toldt’s fascia, which was confused with the Gerota’s fascia of the left kidney. Tight adhesion was found near to the descending section of the duodenum. Following entrance to the potential space of the mesentery by peritoneal incision lateral to the descending colon, the mass was easily separated by blunt dissection. The trunk of the superior mesenteric vessels was carefully protected, and sharp and blunt dissection were practiced to control the tight adhesion in the duodenal region. Visible tumor-related lymph vessels were most concentrated in the superficial layer covering the head of the pancreas and drained towards the retroperitoneum. These lymph vessels were ligated carefully. The mass was removed completely without tumor perforation and intra-abdominal pollution (Fig. 2). The duodenum, small intestine and capsule of the pancreas were checked to confirm that there was no damage. Two drainage tubes were used; tube 1 in the left paracolic gutter and tube 2 near to the descending section of the duodenum above the head of the pancreas.

**Figure 3:**
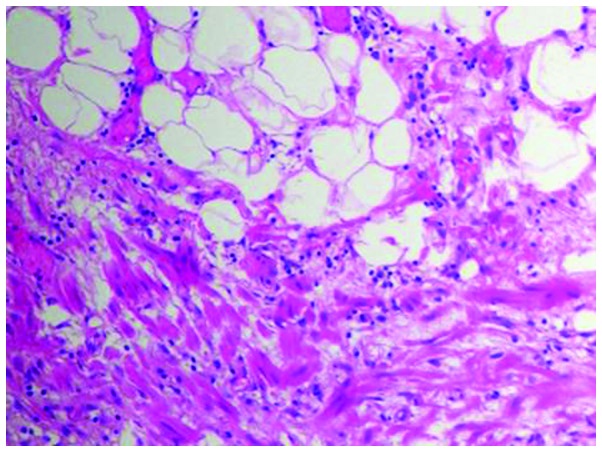
Cystic walls comprised of smooth muscle and flat endothelial cells (hematoxylin and eosin; magnification, ×10).

**Figure 4:**
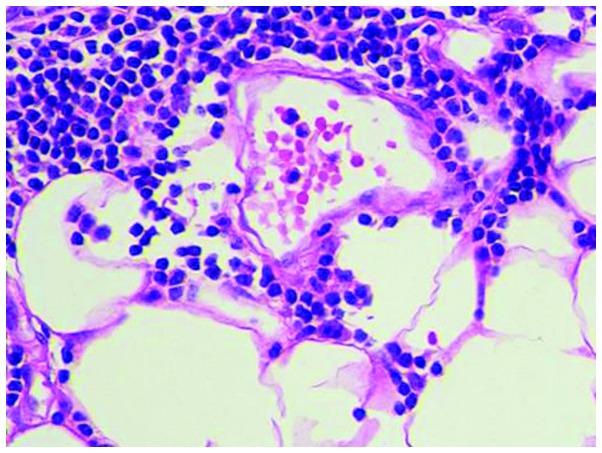
Lymphocyte infiltration of the cystic space (hematoxylin and eosin; magnification, ×40).

Investigation of the resected tumor identified that each lobule was filled with a milky white fluidic content. Following fixation with formalin, the size of the tumor was reduced to 29.5×16.5×3.0 cm. Multicystic structures were found with morphological observation, and the diameter of the cysts ranged between 0.3 and 2 cm. Caseous material remained inside the cysts following drainage of the milky white fluid. Microscopically, the cystic walls of the tumor were comprised of flat endothelial and smooth muscle cells (Fig. 3). Lipid deposition occupied the intracystic space lined by flat endothelial cells and lymphocyte infiltration (Fig. 4). The pathological diagnosis supported lymphatic hygroma.

The patient’s first bowel movement occurred three days after surgery. Drainage tube 1 (in the left paracolic gutter) was removed without any adverse events on day 6. A milky chylous fluid appeared in tube 2 (placed at the descending section of the duodenum), since a fluid diet had been administered on day 4. A chyle test of the drainage indicated a positive result that confirmed the diagnosis of chylous leakage. Since the volume of the drainage was 30–50 ml each day, which is clinically acceptable, no special treatment was provided and a semi-fluid diet was initiated on day 7. The patient recovered well post-operatively and the drainage was removed on day 14 when the drainage volume had decreased to 2–3 ml per day. The patient provided written informed consent.

## Discussion

Lymphangiomas are divided into the following three major histological types: Simple or capillary hemangioma, cavernous hemangioma and lymphatic hygroma (5,6). The cavernous type is most frequently found in the mesenteric region and colonic wall (8). Lymphatic hygromas are defined as malformations of the lymphatic system appearing as single or multiloculated cavities (9). Pathologically, lymphatic hygromas are composed of dilated cysts containing proteinaceous eosinophilic fluid, separated by endothelial cells (10,11). Symptoms occur only after the lymphatic hygroma compresses the adjacent structures (12). The symptoms of abdominal lymphatic hygroma are not specific, including transient pain, nausea, vomiting, abdominal distention, ascites and fever. Complications include inflammation, intestinal or ureter obstructions, cystic ruptures and bleeding (13–15). The most common location of mesenteric lymphangioma is the small bowel volvulus, usually complicated by acute intestinal obstruction (16). In the present case, the mesenteric lymphangioma was located at the small bowel volvulus, but did not cause the complication of intestinal obstruction.

The etiology and pathogenesis of lymphatic hygroma are poorly understood. It has been indicated that its possible etiologies are attributed to chronic inflammation and fibrosis, trauma, lymph node atrophy and lymphatic endothelial disturbances (5). Genetic factors are also important for the formation of lymphatic hygroma, which may lead to the loss of connection between the lymphatic and venous systems, abnormal budding of lymphatic structures from the cardinal vein and abnormal sequestration of lymphatic tissue in early embryogenesis (17). Previously, Chen *et al* (18) showed that the congenital malformation causes sequestration of lymphatic vessels during the embryonic period.

A pre-operative diagnosis of lymphatic hygroma is often difficult, since it is difficult to distinguish lymphatic hygroma from other cystic masses due to its rarity. The radiographic characterizations are profitable for arranging treatments and predicting lower-stage lesion treatment outcomes. The sonographic characteristic of a multilocular lesion filled with clear fluid matches the diagnosis of lymphatic hygroma (19). Lymphatic hygroma typically appears as a large, thin-walled, multiseptate cystic mass on CT scan (20,21). Enhanced CT scans show enhancement of the cyst walls and septa of the lymphatic hygroma (22). In addition, Choi *et al* (23) previously described the use of magnetic resonance in the diagnosis of lymphatic hygroma. A correct diagnosis for lymphatic hygroma is only possible based on these examinations, which complement each other and whose use may not be beneficial alone (15,24).

Multiple staging systems have been developed for lymphatic hygromas. According to histological results, lymphatic hygromas are divided into three categories (5,6). However, such categories do not correspond with the clinical behavior or therapeutic response. Previous studies by de Serres *et al* (25)and Hamoir *et al* (26) demonstrated an additional staging system for lymphatic hygroma, according to the predicted prognosis and surgical results. In addition, Smith *et al* (27) categorized lymphatic hygromas as macrocystic, microcystic or mixed type based on the sclerotherapy outcome. Currently, no standard classification system exists. Further clinical study is required for establishing a suitable classification procedure.

The goal of lymphatic hygroma treatment is the restoration or preservation of functional integrity and the patients’ quality of life. Lymphatic hygromas do not only compress and infiltrate adjacent structures, but occur with complications, including intracystic hemorrhage, cyst rupture, volvulus or infection (28). As a result, surgery is a beneficial therapy for such cases. If no significant functional deficit is identified, treatment may be delayed and consist of surgery, sclerotherapy or observation (29). The current report set the following indications for abdominal lymphatic hygroma: i) Acute abdominal symptoms; ii) large mass affecting patients’ quality of life; iii) intracystic hemorrhage, cyst rupture, volvulus or infection; iv) obstruction of the intestine, colon or urinary system; and v) compression of the main vein or artery. All these factors also contribute to the timing of intervention. In the current case, the lymphatic hygroma was extremely large in size, compressing the adjacent structures and therefore, matched the indication for surgery.

The standard treatment of lymphatic hygroma is surgical excision (1). A complete resection of the lymphatic hygroma and a margin of the healthy tissue must be performed in order to prevent recurrence (8). The surgical approach must take into account that the process warrants preservation of vital structures. Subtotal or partial lymphatic hygroma excision is also common, dictated by special organ infiltration or proximity to neurovascular structures (30). A study by Raveh *et al* (31) showed that incomplete resection may not cause recurrence, requiring additional therapeutic intervention.

The current study presents the case of a 23-year-old male patient diagnosed with mesenteric lymphatic hygroma, with the chief complaint of progressive dull pain in the upper abdominal region that had been present for seven years. A correct diagnosis was possible following combined analysis of a CT imaging examination and routine abdominal ultrasound. Treatment by complete resection was successful and no severe complications occurred. Large mesenteric lymphatic hygromas continue to pose a therapeutic challenge. In the majority of cases, the treatment planning for adults is primarily determined by the presence of functional compromise or associated symptoms. The mainstay of lymphatic hygroma treatment is surgical resection (32,33). Incomplete excision is the only reason for disease recurrence (34). However, whether to use complete or staged surgical excision, sclerosing agents or other therapeutic modalities must be determined by the size, location and characteristics of the lymphatic hygroma. Progress has been made in understanding the etiology, diagnosis and treatment of lymphatic hygroma, however, future studies remain necessary to improve the management of mesenteric lymphatic hygroma.
